# Ultrasound biomicroscopy findings of 25 G Transconjuctival sutureless (TSV) and conventional (20G) pars plana sclerotomy in the same patient

**DOI:** 10.1186/1471-2415-6-7

**Published:** 2006-02-28

**Authors:** Ravi Keshavamurthy, Pradeep Venkatesh, Satpal Garg

**Affiliations:** 1 Dr. Rajendra Prasad Centre for Ophthalmic Sciences, All India Institute of Medical Sciences, New Delhi, India

## Abstract

**Background:**

Transconjunctival Sutureless Vitrectomy (TSV) is a recent advancement in vitreo-retinal surgical techniques involving the use of 25 G instruments through self-sealing sclerotomies. It has been hypothesized that there may be less chance of vitreous and retinal herniation in the scleral wound as compared to conventional sclerotomy incision. However there are no reports on differences in 20 gauge and 25 gauge sclerotomies using ultrasound biomicroscopy (UBM). We report herein the differences in sclerotomies undertaken with 20 gauge (G) and 25 gauge instruments in the same patient.

**Case presentation:**

Ultrasound biomicroscopy of the sclerotomy sites was done in the same patient in whom both 20 G and 25 G sclerotomies had to be constructed during pars plana vitrectomy and the differences were studied.

On day 2, we observed a wide gape at the site that had been enlarged using a 20G MVR blade. In contrast, the other two sites made transconjunctivally using the 25G trocar showed only a mild gape. Significant gape continued to persist at the subsequent evaluations on day 7 and day 14 only at the port, which had been enlarged.

**Conclusion:**

Healing of a 25 G sclerotomy is expectedly quite rapid, with inability to detect the site of sclerotomy in a short duration of 2 weeks post-operatively. This is as opposed to conventional sclerotomies, which might take up to 6–8 weeks post-operatively for complete opposition.

## Background

Transconjunctival Sutureless Vitrectomy (TSV) is a recent advancement in vitreo-retinal surgical techniques involving the use of 25 G instruments through self-sealing sclerotomies. The obvious advantages of such a technique include minimization of surgically induced trauma from sclerotomy sites, less post-operative inflammatory response with faster post-operative recovery [1]. Additionally, it has been hypothesized that there may be less chance of vitreous and retinal herniation in the scleral wound as compared to conventional sclerotomy incision [2]. The healing of sclerotomies is best studied using ultrasound biomicroscopy [3,4]. Previously, comparative study of conventional 20G and sutureless 20G pars plana sclerotomies has been done using ultrasound biomicroscopy [2]. However, there was no difference in the size of the sclerotomy, which was constructed as a self-sealing scleral tunnel. In this report, ultrasound biomicroscopy of the sclerotomy sites was done in the same patient in whom both 20G and 25 G sclerotomies had to be constructed during pars plana vitrectomy.

## Case presentation

A 25 year old male patient was admitted with a diagnosis of Eales' disease and a non-resolving vitreous hemorrhage involving the left eye of 2 months duration. Ultrasound evaluation of the posterior segment revealed findings consistent with an organized vitreous hemorrhage and incomplete posterior vitreous detachment. Transconjuctival sutureless pars plana vitrectomy was planned using the Bausch and Lomb 25G vitrectomy system. Per-operatively, sleeve for the infusion line was first placed in the inferotemporal quadrant transconjunctivally using the 25 G trocar. After inserting the infusion line through the above sleeve, two more sleeves for the endo illuminator and vitreous cutter were made using the 25G trocar transconjunctivally. Vitrectomy was started using high cut rates (1500 cuts/min) and suction of 500 mm Hg. A fibrovascular frond was noted attached to the disc, which had to be diathermised. To accommodate a conventional unimanual bipolar diathermy probe a localized peritomy was made and the superonasal sclerotomy was enlarged using a 20 G MVR blade. The frond was diathermised, endolaser pan retinal photocoagulation was completed and the conventional sclerotomy was closed using 6-0 vicryl mattress suture placed at a depth of about 75% of the scleral lip. Vitreous was well cleared from this wound opening using the vitrectomy probe around the edges of the sclerotomy before closure. The sleeves of the remaining two 25 G sclerotomies were removed and the overlying conjunctiva was slightly massaged in that area. No wound leak was noted at the end of the procedure. Postoperatively intraocular pressure of 10 mm Hg was recorded.

**Figure 1:**
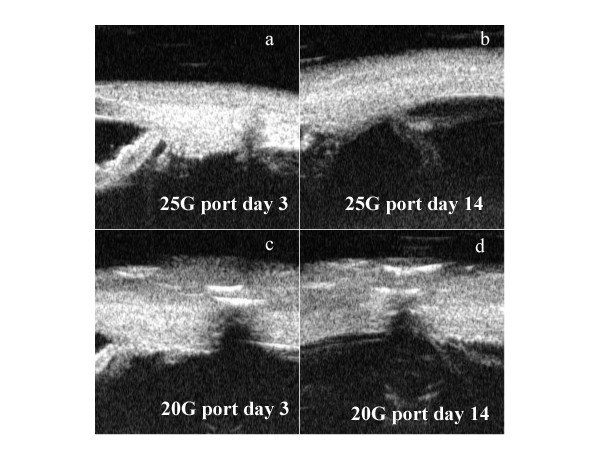
UBM image of 25G port on postoperative day 3 (a) and day 14 (b) shows complete healing of the sclerotomy. Converging vitreous strand is also evident. In contrast there is persisting gape of sclerotomy site enlarged to 20G (in the same patient) on day 3 and 14 (figure c and d).

Ultrasound biomicroscopy (Paradigm model P40) of the sclerotomy sites was done on day 2 postoperatively and again repeated on day 7 and day 14. The port sites were identified at the 20G site by the color of the vicryl suture and at the 25G site by the distortion of the overlying conjunctival vasculature. On day 2, we observed a wide gape at the site that had been enlarged to accommodate the diathermy probe using a 20G MVR blade. In contrast to this, the other two sites wherein sleeves were placed transconjunctivally using the 25G trocar showed only a mild gape (figure 1). While significant gape continued to persist at the subsequent evaluations on day 7 and day 14 only at the port that had been enlarged, the other two ports showed only a minimal gape of the internal lip (figure 2). In addition, we also observed some vitreous incarceration at one of the 25 G sclerotomy sites.

## Discussion

Ultrasound biomicroscopy has in the recent past become an important tool in the management of vitreo-retinal diseases providing cross-sectional images of the peripheral retina, pars plana and the vitreous base, areas which are difficult to visualize adequately, even with indirect ophthalmoscopy and scleral indentation [5,6]. It has been used pre-operatively to assess anterior proliferative retinopathy and plan a proper site for sclerotomy and also post-operatively for early detection of ciliochoroidal detachment after pars plana vitrectomy [5,6].

Boker and Spitznas were the first to report usage of ultrasound biomicroscopy to examine the sclerotomy site after pars plana vitrectomy [3]. A significant finding in their study was the presence of membranous stalks originating from the sclerotomy sites in eyes treated for complications after vitrectomy. Tardif and Schepens examined 10 eyes that had fibrovascular proliferation after diabetic vitrectomy and noted that the proliferation was more common at the active port site and proposed a possible role of introduction of multiple instruments through the same sclerotomy as a cause for the ingrowth [7]. Bhende et al having studied the healing patterns of sclerotomies in patients with proliferative diabetic retinopathy undergoing pars plana vitrectomy classified the changes at sclerotomy site into six groups: well healed, gape, plaque, vitreous incarceration, fibrovascular proliferation and anterior hyaloidal fibrovascular proliferation [4]. They found that the incidence of vitreous incarceration and fibrovascular proliferation was maximum at the infusion port site and cited inadequate vitreous clearance at that site as a cause for proliferation. They also found that in diabetic patients with recurrent vitreous hemorrhage, presence of fibrovascular proliferation at the sclerotomy site was an indicator of a need for an aggressive surgical intervention.

Pars plana vitrectomy in which self sealed 20G sclerotomies have been constructed has been hypothesized to have less chance of vitreous incarceration [2]. However, Kwok et al in their comparative study of conventional and sutureless pars plana sclerotomies using ultrasound biomicroscopy noted no difference in the amount of vitreous incarceration in the two groups.

## Conclusion

Present report documents the natural course of healing of a 25 G sclerotomy in comparison to the healing of a conventional sclerotomy in the same patient. An extensive Pub Med search did not reveal any other report documenting such a comparison. Our observations show that healing of a 25 G sclerotomy is expectedly quite rapid, with inability to detect the site of sclerotomy in a short duration of 2 weeks post-operatively. This is as opposed to conventional sclerotomies, which might take up to 6–8 weeks post-operatively for complete opposition. Decrease in the size of the sclerotomy also seems to have no predictable bearing on the occurrence of vitreous incarceration as observed in our case.

## Abbreviations

UBM: Ultrasound biomicroscopy; TSV: Transconjunctival Sutureless Vitrectomy; G: Gauge; MVR: MicroVitreoRetinal blade.

## Competing interests

The author(s) declare that they have no competing interests.

## Authors' contributions

Ravi Keshavamurthy: Resident involved in patient workup and assistance.

Pradeep Venkatesh: Faculty involved in undertaking UBM and co-coordinating the case report.

Satpal Garg: Chief surgeon.

## Pre-publication history

The pre-publication history for this paper can be accessed here:


